# Quality improvement activity in occupational healthcare associated with reduced need for disability retirement: A Bayesian mixed effects modelling study in Finland

**DOI:** 10.5271/sjweh.3901

**Published:** 2020-10-30

**Authors:** Jarmo Kuronen, Klas Winell, Juho Kopra, Kimmo Räsänen

**Affiliations:** 1 Orchid ID 0000-0001-8929-070X; Etelä-Savon Työterveys Oy, Mikkeli, Finland; 2 Conmedic Oy, Espoo, Finland; 3 Etelä-Savon Työterveys Oy, Faculty of Health Sciences, School of Medicine, Institute of Clinical Medicine, Kuopio Musculoskeletal Research Unit, Kuopio, Finland; 4 University of Eastern Finland, Faculty of Health Sciences, School of Medicine, Institute of Public Health and Clinical Nutrition, Kuopio, Finland

**Keywords:** alcohol, depression, disability pension, health check-up, networking, pension, work ability

## Abstract

**Objectives:**

There is evidence that occupational healthcare (OHC) may improve employees’ work ability. This research was designed to study whether common quality improvement (QI) activities in the OHC quality network (OQN) – a voluntary collaborative forum – can reduce the need for disability pensions.

**Methods:**

The study population comprised employees under the care of 19 OHC units in Finland affiliated with the OQN. The association of 12 QI activities with new disability pensions during the years 2011–2017 was analyzed by Bayesian mixed effects modelling.

**Results:**

Patients of OHC units affiliated with the OQN have fewer full permanent disability pensions [odds ratio (OR) 0.77, 95% credible interval (CI) 0.60–0.98] and full provisional disability pensions (OR 0.68, 95% CI 0.53–0.87) than patients of unaffiliated units. Of the studied QI activities, the measurements of intervening in excessive use of alcohol had the strongest association with the incidence of all disability pensions (OR 0.53, 95% CI 0.41–0.68). Participation in the focus of work measurements and quality facilitator training was also associated with the reduced incidence of disability pensions (OR 0.84, 95% CI 0.71–0.98, and OR 0.92, 95 CI 0.84–0.99, respectively).

**Conclusions:**

Affiliation with a quality network seemed to improve outcomes by reducing full disability pensions or replacing them by partial disability pensions. Some QI activities in the OQN were associated with a reduction of disability pensions.

Efforts are underway globally to improve the quality, coverage and security of healthcare (1). Quality improvement (QI) initiatives in healthcare aim at enhancing service delivery through process development (2). Such initiatives include activities that strive to improve existing processes or redesign processes altogether (2–4). Occupational healthcare (OHC) should focus on its core tasks, which are the promotion of healthy workplaces (5) and ensuring the health and work ability of employees (6, 7).

Processes intended to enhance and maintain employees’ work ability should be established on evidence-based methods. Initiatives designed to reduce the risk of work-related disability can be particularly effective. These include general job modification, return-to-work coordination and organizational support (5), and targeted health check-ups for employees with work incapacities, followed by health-focused and workplace interventions addressing cases of musculoskeletal and mental disorders (8, 9). Reorganization of OHC services, including service coordination and rehabilitation after injuries, has been shown to reduce disability pensions (10). Multi-domain interventions in the workplace including work adjustments, changes of role or work tasks, reductions in working hours, sabbaticals, physiotherapy and physical exercise may reduce the likelihood of disability pension (11). Early vocational rehabilitation can have the same effect (12). While depression is one of the main global reasons for disability, in Finland improvement and better organization of care are required before disability pension is granted due to depression (13, 14).

Disability retirement is predominantly caused by mental and musculoskeletal disorders (15). Other causes include cardiovascular diseases and accidents (15). Due to the ageing of the labor force (16), a lot of emphasis is put on the promotion of work participation and avoidance of early retirement (17). Predictive risk estimates have been developed for the prevention of chronic conditions (18). Once preventive activities have been implemented in OHC it can take a long time, even decades, for effects to be seen (19, 20). This means that outcome measures often cannot be used, thus process performance indicators are preferred in improvement measurements (19).

Often the results of OHC are assessed only by the resources employed and the number of patient visits because these are easy to measure. Instead, we should strive to measure the processes and their outcomes (eg, the reduction in the disability retirement). Measuring the performance of a process is more demanding and expensive than measuring the number of patient visits (2, 21) but gives more evidence of its effectiveness (4). Data on the performance of healthcare processes can be used to improve both the organization of care and clinical decision-making (22).

In addition to the evidence that OHC may improve the prevention of disabilities (8, 23) there is also evidence that networking may improve the quality and outcomes of healthcare, although this has not been studied in the field of OHC (24).

One of the major shared goals of employers, employees and OHC is the reduction of early retirement due to disability. Although much has been written about QI in healthcare, little is known about its impact in OHC on disability retirement. Therefore, we designed a study to find out whether the implementation of common QI activities in the occupational healthcare quality network (OQN) – a voluntary collaborative forum consisting of OHC service providers – reduces the need for disability retirement and which specific activities affect the outcomes. The study hypothesis was that common QI activities in the OQN are effective in reducing disability pensions.

## Methods

### The occupational healthcare quality network

At its foundation in 2011, the Finnish OQN had two goals: to reduce the rate of disability retirement, especially the full permanent disability pension, and lower the number of full disability pensions in favor of partial pensions. Conmedic, a nonprofit company that coordinates healthcare quality networks and facilitates the OQN, performed the data analyses of quality measurements. The members of the OQN support each other’s development by exchanging material and information on best practices. Each OHC unit consists of occupational health physicians, occupational nurses, occupational physiotherapists and occupational psychologists. Since the foundation of the OQN, each unit has performed several of the common QI activities. The member units of the OQN have used the continuous quality development model in the development of processes (25) with repeated measurements of process indicators. This study included activity data from 19 OHC units across Finland that have been affiliated with the OQN either through the whole study period or for only a few years.

### The study population and data

The outcome data covered the disability pensions of all Finnish municipality employees during 2005–2017 obtained from the pension registers of Keva, the largest pension provider in Finland which administers the pensions of public sector workers. The clients of the OHC units (municipalities) had to agree to the retrieval of data on disability retirements among their employees from Keva records. All the data from Keva were aggregated and anonymous with no possibility for an individual’s identification.

The activity data were collected from January 2011 to December 2017, during which time the subset of OHC units affiliated with the OQN has varied. Regardless, the activity data of all participants was collected.

The study population consisted of all employees in the care of the 19 OHC units affiliated with the OQN between 2011 and 2017. The comparison population were all municipality employees in the care of non-affiliated OHC units. The annual number of employees in the study population ranged between 32 239–38 438 and 445 628–487 921 in the comparison population during the period 2005–2017.

The 12 QI activities analyzed were: participation in (i) measurements of intervening in excessive use of alcohol; (ii) quality measurements of health check-ups; (iii) quality facilitator training; (iv) focus of work measurements; (v) resource measurements; (vi) quality network workshops; (vii) peer review training; (viii) quality measurements of depression care; (ix) advisory board of the OQN; (x) employee and employer satisfaction surveys; and reporting of (xi) QI plans for the coming year; and (xii) QI activities.

The focus of work measurements included measuring the following indicators in registers: proportion of the work done in preventive healthcare (following the classification of the National Pension Institution), proportion of all contacts directed to employees with musculoskeletal or mental disorders (which are the main reasons for early retirement), and the number of tripartite negotiations (employee with reduced work ability, employer and OHC) per year. The QI activities with their descriptions are presented in table 1.

**Table 1:** Definition of affiliation with the Finnish Occupational Health Quality Network (OQN) and the 12 quality improvement activities. [OHC=occupational healthcare.]

Quality improvement activity of the OQN	Definition
Affiliation with the OQN	The OHC units that have been affiliated with the OQN either through the whole study period or only a few (2–4) years.
Participation in measurements of intervening in excessive use of alcohol	Number of times the OHC unit has participated in the quality measurement of performing a brief intervention with employees who have excessive use of alcohol. Yearly 2-day measurement has been performed three times. The measurement with 17 indicators directs to all consultations in the OHC.
Participation in health check-up quality measurements	Number of times the OHC unit has participated in the yearly quality measurement of health check-ups. Consists of all consecutive health check-ups targeted to employees with reduced working capacity or hazardous work (31 indicators) and performed by any of the professionals in the OHC unit (OHC physician, OHC nurse, OHC physiotherapist or OHC psychologist) during a 2–4 week time (depending on the size of the OHC unit).
Participation in quality facilitator training	Number of persons that have participated in the training. The training consists of three 2-day educational sessions with development tasks in the own OHC unit between the sessions.
Participation in focus of work measurements	Number of times the OHC unit has participated in the yearly focus of work measurement. The measurement consists of patient flow and billing data of the OHC unit (6 indicators of proportion of preventive work, proportion of consultations directed to employees with musculoskeletal or mental problems and number of three party negotiations with employee with reduced work ability, employer and OHC).
Participation in resource measurements	Number of times the OHC unit has participated in the yearly resource measurement which consists of personnel structure and finances of the OHC unit (14 indicators).
Participation in quality network workshops	Number of persons, who have participated in the workshops, which consist of two or three educational days per year.
Participation in peer review training	Number of persons who have participated in the training which consists of coaching the OHC team to run a structured peer review, perform one and be target for one.
Participation in quality measurements of depression care	Number of times the OHC unit has participated in the yearly quality measurement of depression care. The measurement consists of 50 or more previous patients diagnosed with depression (35 indicators).
Participation in advisory board of the OQN	Number of times the leaders of the OHC unit have participated in the board meetings. The advisory board meets once a year to determine the activities of the OQN for the incoming year.
Participation in employee and employer satisfaction surveys	Number of times the OHC unit has participated in the yearly satisfaction measurement of employees and employers. The measurement consists of questionnaires to all client companies of the OHC unit (50 indicators).
Reporting the quality improvement plans for the coming year	Number of times the OHC unit has announced the improvement plans. Consists of the main activities to come of the OHC unit.
Reporting quality improvement activities	Number of times the OHC unit has participated in the yearly reporting. The report consists of all quality improvement activities performed during the previous year.

We used the incidence of different disability pensions as outcome measures for the effects of QI activity in the OQN. The incident disability retirement was calculated by dividing the number of granted disability pensions by the number of insured employees at the end of each year. The definitions of the different pension benefits are described in table 2. We followed the STROBE guideline in the set-up of the study.

**Table 2:** Definitions of outcomes.

Outcome indicator	Definition
Full permanent disability pension	An individual must have a permanent reduction in the work capacity of over 60%.
Partial permanent disability pension	An individual must have a permanent reduction in the work capacity of 40-60%.
Full provisional disability pension	An individual must have a temporary reduction of the work capacity over 60%. Usually granted for no more than one year during which time the person is rehabilitated.
Partial provisional disability pension	An individual must have a temporary reduction of the work capacity of 40-60%. Usually granted for no more than one year during which time the person is rehabilitated.

### Statistical analysis

The statistical analysis was run by using a Bayesian generalized linear mixed model consisting of both fixed and random effects (26). The mixed-effect approach was chosen because it allows the modelling of employer-specific variability and yearly variability of data, thus rendering estimates that are representative of the overall effect of each fixed effect covariate of interest.

As there was no separate reference group to compare the activity data with, the units not affiliated with the OQN each year were used in the statistical model to estimate the effect of the covariates to the outcome. The random effects take care of adjusting for yearly variability as well as OHC-unit-specific variability, so that the fixed-effects estimates represent the overall effect of the study population.

Credible intervals (a Bayesian analogy to confidence intervals) are reported. Readers with no Bayesian background can follow the results in table 3 by interpreting the 95% credible intervals (95% CI) similarly as confidence intervals for odds ratios (OR).

**Table 3:** The odds ratios (OR) with 95% credible intervals (CI) for disability pensions of affiliation with the quality network and participation in quality improvement (QI) activities (covariates) of the occupational healthcare units. [OQN= Occupational Health Quality Network; QM=quality measurement; ns=the independent covariate has been excluded from the final model due to model selection.]

Covariate	Full permanent disability pension OR (95% CI)	Partial permanent disability pension OR (95% CI)	Full provisional disability pension OR (95% CI)	Partial provisional disability pension OR (95% CI)	All disability pensions OR (95% CI)
Affiliation with the Finnish OQN	0.77 (0.60–0.98)	0.82 (0.58–1.13)	0.68 (0.53–0.87)	2.42 (1.41–3.84)	0.86 (0.72–1.03)
Participation in					
QM of intervening in excessive alcohol use	ns	0.35 (0.22–0.55)	0.69 (0.48–0.96)	0.60 (0.29–1.14)	0.53 (0.41–0.68)
QM of health check-ups	ns	0.74 (0.57–0.95)	ns	0.59 (0.39–0.85)	0.87 (0.75–1.00)
Quality facilitator training	ns	0.92 (0.78–1.07)	ns	ns	0.92 (0.84–0.99)
Focus of work measurements	ns	0.77 (0.57–1.02)	ns	1.21 (0.75–1.85)	0.84 (0.71–0.98)
Resource measurements	0.77 (0.53–1.11)	1.36 (0.99–1.83)	ns	ns	ns
Quality network workshops	ns	1.09 (1.02–1.16)	1.06 (1.01–1.11)	1.03 (0.94–1.13)	1.05 (1.02–1.09)
Peer review training	ns	1.12 (1.04–1.20)	ns	1.05 (0.93–1.18)	ns
QM of depression care	ns	1.40 (0.99–1.91)	ns	1.27 (0.77–2.00)	1.27 (1.05–1.51)
Advisory board of the quality network	ns	ns	1.33 (0.98–1.76)	1.43 (0.67–2.67)	1.46 (1.17–1.81)
Employer & employee satisfaction measurements	ns	ns	ns	ns	ns
Reporting					
QI plans for the coming year	ns	1.53 (1.02–2.21)	ns	2.11 (1.13–3.65)	1.34 (1.09–1.62)
QI activities	ns	1.30 (0.86–1.87)	ns	ns	ns

The Bayesian model was implemented using Just Another Gibbs Sampler (JAGS) software (27) and data preprocessing was done in R (28). Each model was fitted to the data using four Markov Chain Monte Carlo (MCMC) chains (29) with a total length of 500 000 iterations. The first 20 000 iterations were discarded as a burn-in. Thinning to every 100 iterations were used leading to 19 200 iterations stored for each model. The MCMC chains appear to have converged based on both visual inspection and Brooks-Gelman-Rubin Rhat-diagnostics (30). The Rhat-values were <1.006 for all covariates in the model. The Rhat-value 1.00 indicates the perfect convergence and all values <1.05 are acceptable.

*Description of the model*. The data has *N* observations where the index *i* runs over the rows of data. The response *y_i_ = (k_i_,n_i_)* consists of the number of disability retirements *k_i_* and number of employees *n_i_* in OHC care during the year at row *i*. In addition, we utilized the 12 QI activities as indicators of participation in the OQN. We included the covariate “Affiliation with the Finnish Occupational Healthcare Quality Network”, which indicates whether the OHC *Emp(i)* participated in any QI activity during *Year(i)*, which also defines it as being affiliated with the OQN.

We generated five different models (marked #), each with different response covariates, but all within the same modelling setting. For each model, the response *y_i_ ~Binom (p_i_,k_i_)* stands for #1 Full permanent disability pension, #2 Partial permanent disability pension, #3 Full provisional disability pension, #4 Partial provisional disability pension or #5 All granted disability pensions.

The probability *P(y_i_ = 1) = p_i_* of obtaining a disability retirement was modeled by the following equation:


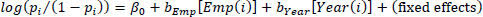


In this equation, the parameter *ß_0_* is a constant that defines the base level for retirement. Parameters *b_Emp_* and *b_Year_* are random effects for employer and calendar year, and the term *fixed effects* depends on the model. The presence of the random effect term *b_Emp_ [Emp(i)]* factors in this model means that the measurements from the same employers in different years are more similar to each other than to those measured among the other employers. Respectively, *b_Year_ [Year(i)]* factor is the year-dependent effect of different years. The random effect terms follow Gaussian distribution:


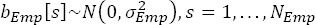


and


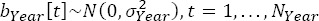


*Prior probability distributions*. The prior probability distributions, which represent information regarding model parameters available prior to the analysis were as follows: For the constant and each fixed effect parameter, the *N(0,1000)* was used. For the variance parameters 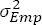 and 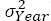 of the random effects 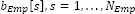 and 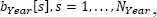, the inverse gamma distribution with parameters 0.001 and 0.001 was used.

*Model selection*. The models were selected according to the deviance information criteria (DIC) (31). After the model selection phase, each of the selected five models had a different number of covariates remaining. The model coefficients are transformed to OR using *OR = exp(β)* where *ß* stands for fixed effect. The OR for fixed effects of all models are reported in table 3. Where a numerical value is not given (ns) the independent covariate has been excluded from the final model due to model selection. For example, in the model of “Full permanent disability pension”, the only remaining fixed effects are affiliation with the Finnish OQN and participation in resource measurements.

## Results

**Figure 1:**
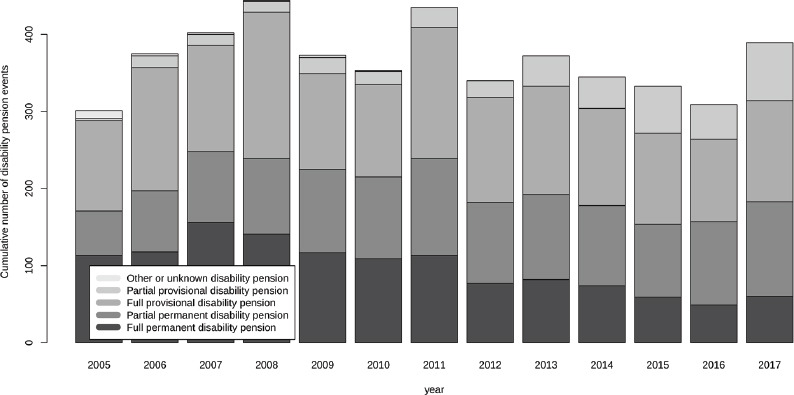
The cumulative number of different categories of granted disability pensions in the study population by year from 2005 to 2017 showing the development even before the study period 2011-2017.

The mean age of the employees who were granted a disability pension in 2017 was 55.1 years in the study population while it was 54.1 years among all municipality employees. The number of different disability pension types granted during 2011–2017 are shown in figure 1. The number of full permanent disability pensions decreased from 31.7 grants /10 000 employees in 2011 to 13.6 grants /10 000 employees in 2017, while partial permanent disability pension and partial provisional disability pensions had a slightly increasing trend. The number of provisional pensions granted fluctuated during the period and peaked in 2008.

The affiliation of OHC units with the OQN is associated with a reduction in full disability pensions compared to the unaffiliated units, OR for full permanent disability pension 0.77, 95% CI 0.60–0.98 and for full provisional disability pension 0.68, 95% CI 0.53–0.87 (table 3). For all disability pensions, the association was not as strong as it was with full permanent disability pensions (OR 0.86, 95% CI 0.72–1.03).

Some of the QI activities appeared to be especially valuable in reducing disability retirement. Participation in the quality measurements of intervening in excessive use of alcohol was strongly associated with the reduction of all disability pensions (OR 0.53, 95% CI 0.41–0.68). The association with the partial permanent disability pensions was even stronger (OR 0.35, 95% CI 0.22–0.55). Participation in the quality measurements of health check-ups (HCU) was associated with the reduction of all disability pensions (OR 0.87, 95% CI 0.75–1.0). The association was especially strong with both types of partial disability pensions. Participation in the focus of work measurements associated also positively with the reduced incidence of total disability retirement (OR 0.84, 95% CI 0.71–0.98) as did participation in the quality facilitator training (OR 0.92, 95% CI 0.84–0.99) (table 3).

Some QI activities had negative associations with the reduction of incidence in disability pensions. Participation in the quality measurements of depression care was associated with an increased risk of total granted disability pensions (OR 1.27, 95% CI 1.05–1.51), participation in quality network workshops increased the OR, as did reporting the QI plans for the coming year and participating in the advisory board meetings (table 3).

The random effect variation was higher for employers (municipalities) random effects than for yearly random effects for all the models. The standard deviations for random effect of employers were 0.415 (95% CI 0.266–0.628) in full permanent disability pension, 0.312 (95% CI 0.197–0.480) in partial permanent disability pension, 0.251 (95% CI 0.139–0.416) in full provisional disability pension, 0.551 (95% CI 0.276–0.873) in partial provisional disability pension and 0.264 (95% CI 0.172–0.400) in all disability pension together. The standard deviations for random effects of the study year were 0.241 (95% CI 0.119–0.426), 0.124 (95% CI 0.037–0.247), 0.144 (95% CI 0.072–0.25), 0.185 (95% CI 0.031–0.474), and 0.116 (95% CI 0.064–0.196), respectively. During the model selection process, covariates were dropped out from the model one at the time. Table 3 reports the OR for covariates included in the final models. DIC was used for model selection and final values are 957.3, 970.3, 1052.5, 564.6 and 25746.4, respectively.

## Discussion

Our study showed that some QI activities had a positive association with the core outcome of OHC, namely reduction of early disability retirement. We found several improvement activities that were positively associated with work ability but also such that had negative associations. The activities with the strongest positive association with the reduction of early disability retirement were, in the order of their strength, participation in: (i) the quality measurements of intervening in excessive use of alcohol, (ii) the focus of work measurements and (iii) the quality facilitator training.

The affiliation of the OHC unit with the OQN showed a risk reduction of full permanent disability pensions and full provisional disability pensions while partial disability pensions did not differ from the comparison group. These findings might show that some full disability pensions have been replaced with partial disability pensions. We think that the results showed a change towards the intended targets of the OQN, namely, a reduction of full permanent disability pensions and shift to partial disability pensions.

Shifts from full to partial disability benefits, even in short-term sickness absences, have been shown to increase long-term work participation (32) and to bring social security savings (33). The shift towards partial disability benefits can be a way to break a downward spiral pattern found by a Danish study, in which a previous sickness absence was associated with the increased risk of work disability pension through a transition cycle: from work to sickness absence, from sickness absence to unemployment or to a disability pension (34).

We demonstrated a reduction in the granted disability retirements. This positive result may have been driven by the sum of all the QI activities, although it seems that some improvement activities have stronger effects than others. Earlier studies on the reduction of the need for disability retirement have shown the importance of broad multi-domain activities in the workplace in collaboration with OHC (10, 11). Such positive shifts often need multidisciplinary collaboration. In their systematic review, Cullen et al (8) showed that workplace-based return-to-work interventions and work disability management interventions helped workers with musculoskeletal and pain-related conditions and mental health conditions to return to work (8). The authors recommend that the interventions should include healthcare provision, service coordination and work accommodation components.

The QI activities affected the four disability pension types in different ways. The novel and surprising finding of this study was that participation in the quality measurements of intervening in excessive use of alcohol had such a strong association with the reduction of all disability pensions. One may speculate about the reasons why this activity was so effective. One explanation could be that healthcare workers find it difficult to discuss alcohol use. By participating in this activity, the management showed to the OHC personnel that intervening in risky use of alcohol is important. Studies have shown that excessive alcohol use is a strong driver of early retirement in Finland (35, 36). Screening, health advice and support to tackle alcohol-connected risk behavior are inexpensive especially when compared to disability retirement (37). There is also evidence that intervention at the workplace is effective in reducing problems related to excessive alcohol consumption (38).

Participation in the focus of work measurements, such as how diagnoses affect the patient flow, was associated with reductions in all types of disability pensions together. Such measurements can be difficult to make because the data are hard to find in patient and administrative records. The results regarding this activity may reflect this barrier and could show that units willing to perform the activity are also willing to make the necessary changes in patient flow.

The positive association we found concerning participation in the quality facilitator training was anticipated and could reflect the fact that deeper knowledge of the fundaments of QI may bring better results (39). Participation in the quality measurements of HCU had a weak association with the reduction in disability retirement. The reductions were mainly seen in the partial disability pensions. In Finland, HCU in OHC are targeted at employees who are in danger of prolonged sick leave and early retirement, those who are exposed to work hazards (such as chemicals and noise), and controls in 5- to 10-year intervals of the ageing. The first two types were the target group of our study. The associations we found may indicate that the activity has not led to major improvements in the processes that support work ability. However, some studies have found HCU to be a valuable component of Finnish OHC. The targeted HCU may work as triggers for interventions to address poor work ability (9, 40) while they can also work as interventions themselves (8). The health education given during HCU may have some effect in the prevention of occupational asthma and accidents (41). Moreover, health education may be even more important from the point of view that the risk factors found during HCU have predictive value of mortality for decades (20). On the other hand, while targeted HCU seem to be important, untargeted HCU in primary healthcare seem to have little or no effect on mortality or morbidity (42).

It appears that participation in the quality measurements of depression care was associated with an increased risk of all disability pensions. This finding is somewhat surprising since one would expect this measurement activity to show comprehensive interest in the mental health of employees. The result may, however, indicate an excessive presence of mental problems and could reflect the findings of the systematic review of Cullen et al (8), which found that the traditional cognitive therapy methods used in OHC may be ineffective, and highlights the need for new work-focused cognitive behavioral interventions (8). Participation in quality network workshops, reporting the QI plans for the coming year, and participating in the advisory board meetings were also negatively associated with the reduction of disability pensions. It is important to recognize that not all changes result in improvement (4), but perhaps these activities do not have direct influence on the focus of work in the OHC unit.

The strengths of our study were that we were able to evaluate the association of several QI activities with the outcomes – ie, early disability retirement – and follow over a long period the main outcome of effective OHC, namely the prevention of early retirement. It seems that Bayesian mixed-effect modelling can be a feasible method in assessing the effect of multiple QI activities in healthcare.

The study had also weaknesses. Only the common QI activities of the OQN were included in the study. Several improvement activities of OHC based only in one unit were not included in the study. We did not include the results of resource or process measurements in the analysis of this study because of their high number. The OHC units have also participated in them at different times. Other limitations of this study were that the OHC units affiliated with the OQN may have had better OHC practices and greater interest to QI than the non-affiliated units even before the study. This could affect the results per se.

It is possible that the results of our study are not generalizable to all OHC units, not even within Finland. However, the results show the direction for further studies.

### Concluding remarks

This study showed that OHC units reduced the numbers of disability pensions when affiliated with the quality network. Moreover, participation in the quality measurements of intervening in excessive use of alcohol, quality facilitator training, and the focus of work measurements may decrease the risk of work disability pension among employees of companies in the care of OHC units participating in these activities.
